# The Role of Dosimetric Parameters in Radiation Pneumonitis: A Functional Approach in Adjuvant Treatment of Malignant Pleural Mesothelioma

**DOI:** 10.3390/cancers18030405

**Published:** 2026-01-27

**Authors:** Luca Dominici, Davide Franceschini, Mauro Loi, Ruggero Spoto, Antonio Marco Marzo, Beatrice Marini, Mariya Boyanova Ilieva, Nicola Lambri, Francesco La Fauci, Ciro Franzese, Marta Scorsetti

**Affiliations:** 1 Department of Radiotherapy and Radiosurgery, IRCCS Humanitas Research Hospital, Via Manzoni 56, Rozzano, 20089 Milan, Italyruggero.spoto@humanitas.it (R.S.); antonio.marzo@humanitas.it (A.M.M.); beatrice.marini@humanitas.it (B.M.); mariya.ilieva@cancercenter.humanitas.it (M.B.I.); nicola.lambri@humanitas.it (N.L.); francesco.lafauci@humanitas.it (F.L.F.); ciro.franzese@hunimed.eu (C.F.); marta.scorsetti@hunimed.eu (M.S.); 2 Department of Radiation Oncology, Azienda Universitaria Ospedaliera Careggi, 50134 Florence, Italy; mauro.loi82@gmail.com; 3 Department of Biomedical Sciences, Humanitas University, Via Rita Levi Montalcini 4, Pieve Emanuele, 20090 Milan, Italy; 4 Scuola di Specializzazione di Fisica Medica, Università Degli Studi di Milano, 20133 Milan, Italy

**Keywords:** malignant pleural mesothelioma, radiation pneumonitis, dosimetric parameters, functional lung volume, radiotherapy toxicity, V20, IMRT, biomarkers

## Abstract

Background: Malignant pleural mesothelioma (MPM) is an aggressive neoplasm, the major cause of which is asbestos exposure. Adjuvant radiotherapy after pleurectomy/decortication (P/D) aims at reducing locoregional recurrence but is limited by the risk of radiation pneumonitis (RP). In this study, we attempted to evaluate the predictive value of conventional and functional dosimetric parameters in assessing RP risk. Methods: This retrospective study analyzed 68 patients with non-metastatic MPM treated with adjuvant radiotherapy after P/D. Dosimetric parameters, including V20, V5, and mean lung dose (MLD), were calculated for both total lung volume and functional lung volume (FLV), with emphysematous regions excluded based on CT imaging thresholds. Statistical analyses assessed correlations between these parameters and acute RP incidence. Results: Acute RP developed in 42% of patients, and 28% had moderate-to-severe (Grade 2–3) events. V20 and FCL_V20 were significantly associated with the risk of RP (*p* = 0.017 and *p* = 0.028, respectively). Predictive accuracy for conventional V20 (AUC = 0.668) and Functional Contralateral Lung V20 (FCL_V20) (AUC = 0.655) showed moderate efficacy, without further significant improvement in using functional parameters. A V20 threshold > 1.8% predicted severe RP with high specificity (89.8%). Conclusions: While functional lung delineation provides an alternative in dosimetry, conventional V20 is a robust predictor of RP. Optimization of dosimetric constraints, in an effort to reduce pulmonary toxicity in MPM patients, should be further combined with advanced radiotherapy techniques and biomarkers.

## Simple Summary

Malignant pleural mesothelioma (MPM) is a very aggressive cancer that is quite rare and is mostly linked to asbestos exposure. In the case of MPM patients undergoing lung-saving surgery (pleurectomy/decortication), radiation therapy forms an integral part of the treatment plan. However, its use is often limited because of the danger of lung toxicity, especially radiation pneumonitis (RP). It is of utmost importance to discover reliable predictors of radiation pneumonitis that can be utilized for personalizing the treatment plan. This retrospective study was conducted to evaluate the prognostic power of conventional dosimetric parameters—the so-called “functional” parameters which are determined by the exclusion of the emphysematous regions of the lungs—in a group of MPM patients receiving adjuvant intensity-modulated radiation therapy. The results in our hands suggest that functional parameters might offer additional value over standard parameters.

## 1. Introduction

Malignant pleural mesothelioma (MPM) is a rare and very aggressive neoplasm, almost exclusively due to asbestos exposure [1]. Despite the development of multimodal therapeutic strategies, the prognosis remains poor, with limited survival rates. Pleurectomy/decortication (P/D) has become one of the critical surgical interventions for patients with non-metastatic disease, to preserve pulmonary function but allow for complete macroscopic tumor resection.

P/D is invariably followed by adjuvant chemotherapy with a platinum–pemetrexed regimen to achieve better systemic control [2,3,4].

The role of adjuvant radiotherapy has evolved significantly over the past few years [5].

The benefit of this treatment in reducing locoregional recurrence is, however, limited by the risk of severe pulmonary toxicity, particularly radiation pneumonitis (RP) [6,7]. Advanced techniques, such as intensity-modulated radiotherapy (IMRT) and helical tomotherapy, have been shown to reduce exposure to normal lung tissue, thus decreasing the incidence of severe RP [6,8,9,10,11].

Numerous studies have looked into the dosimetric parameters and biological variables influencing pulmonary toxicity. Important predictors, such as the percentage of lung tissue receiving at least 20 Gy, known as V20, and mean lung dose, MLD, have been associated with an increased risk of developing radiation pneumonia [6,8,12,13]. In addition to these, genetic predispositions to radiation-induced damage, as pointed out in most recent studies, suggest that the individualized approach in radiotherapy might further reduce associated risks [6,14,15].

The purpose of this study is to contribute to the existing evidence by analyzing dosimetric and functional parameters related to acute radiation pneumonitis in MPM patients treated with P/D followed by adjuvant radiotherapy. A comparison between conventional and functional lung dosimetry is made in the hope of identifying strategies that enhance safety while preserving therapeutic efficacy.

Further research has also pointed out the utility of functional imaging for enhancing the prognostic efficacy of radiotherapy treatment planning. Their findings support that incorporation of functional lung data might more inform the setting of dose limitations, thereby decreasing the possibility of radiation-induced toxicities in chest cancers [16].

The null hypothesis of this analysis is that functional dosimetric variables offer no advantage in risk prediction of radiation pneumonitis over conventional variables, while the alternative hypothesis is that variables derived from functional contralateral lung (FCL) offer greater predictive ability.

## 2. Materials and Methods

### 2.1. Study Design and Patient Population

This is a retrospective study including patients with non-metastatic MPM treated at a single center with adjuvant radiotherapy after P/D from April 2013 to February 2022. All consecutive patients undergoing surgical resection with the intent of macroscopic tumor reduction, achieving an R0 or R1 resection status, were included in the present study. All patients had histologically confirmed MPM, were in good general condition with an ECOG performance status ≤2, and met the standard eligibility criteria to receive radiotherapy [Table 1].

All patients included in the study had signed a general informed consent accepting the retrospective use of their clinical and dosimetric data for research purposes, in compliance with the institutional policy of our center.

Data on pre-existing pulmonary comorbidities were not systematically available for all patients and were not included in the analysis.

### 2.2. Radiotherapy Protocol

After P/D, patients received adjuvant radiotherapy with the intention of minimizing the risk of local recurrence. Conventionally, irradiation was given in a fractionation scheme with an accumulated total of 44 Gy and subsequently boosted with a further additional dose for a median of 50.6 Gy over the total dosing range from 50.6 to 59.4 Gy at 1.8 to 2 Gy/fraction. Techniques of intensity-modulated radiation therapy were used and optimization for this mode of therapy was carried out, i.e., sparing and, where possible, reducing critical OARs, especially exposure of the contralateral lung.

### 2.3. Delineation of Functional Lung Volume

For assessment and prediction of lung toxicity an original approach was made by delineation of functional lung volume (FLV). The FCL was defined by excluding the emphysematous lung tissue from the dose calculation. Emphysematous regions were identified and excluded based on computed tomography (CT) with a threshold of less than −860 Hounsfield units (HUs), representing the non-functional lung areas often associated with COPD [17,18]. Dosimetric parameters were then recalculated specifically for FCL, and parameters such as V20, V5 and mean lung dose (MLD) were derived for both total lung and functional lung volumes.

### 2.4. Dosimetric Analysis

The standard parameters, V20, V5, and MLD, were calculated for the contralateral lung and functional contralateral lung to assess their correlation with dosimetric parameters and radiation-induced lung toxicity. V20 and V5 indicate the percentage of lung volume through which at least 20 and 5 Gy are transmitted, respectively, whereas MLD indicates the mean dose received by the lung tissue. Comparative analysis was carried out for the purpose of showing a statistical difference between traditional dosimetric values and dosimetric values obtained with functional lung delineation.

### 2.5. Statistical Analysis

There was a test of hypothesis on the relation between the frequency of acute radiation pneumonitis and dosimetric parameters. Parameters of median dose regarding the contralateral lung and functional lung were matched by the test of Wilcoxon signed rank. Finally, the dose–toxicity relationships were examined with Kruskal–Wallis testing for the parameters that significantly correlated with acute toxicity. The receiver operating characteristic curves were drawn, and the Youden index J was calculated in order to investigate the predictive power of each parameter for radiation pneumonitis. A Youden index of greater than 1.4 was considered as a threshold with the highest sensitivity and specificity.

### 2.6. Outcome Measures

The primary endpoint of the study was the incidence of acute radiation pneumonia, graded in accordance with the CTCAE version 4.03. All patients underwent PFTs, clinical examination, and imaging studies to monitor the signs of pneumonia within the first six months after radiotherapy. Secondary endpoints included the evaluation of predictive accuracy of functional lung delineation as compared to conventional dose–volume metrics in predicting acute toxicity.

## 3. Results

The study series consisted of 68 patients with non-metastatic malignant pleural mesothelioma treated at our center with adjuvant radiotherapy after P/D. The median age was 65 years, with patients aged between 37 and 78 years, the majority being males. The majority of the cohort, 85% of them, achieved R0 resection status, meaning no residual tumor post-surgery, while the remaining patients had R1 resection, meaning minimal residual disease (Table 1). Before starting radiotherapy, nearly all patients (96%) received a standard chemotherapy regimen of platinum and pemetrexed, with the number of cycles varying between three and nine per patient. Radiation therapy given was conventionally fractionated to 44 Gy, with an additional boost in 88% of cases to a cumulative dose of 50.6 Gy (range: 50.6 to 59.4 Gy) [Table 1]. This treatment was highly individualized, with advanced IMRT techniques that aimed for optimal tumor control with the sparing of surrounding normal tissue, especially the contralateral lung.

In the dosimetric analysis for the contralateral lung, median values of key parameters were as follows: V20 (volume receiving at least 20 Gy) was 0.5%, V5 (volume receiving 5 Gy) was 55%, and mean lung dose (MLD) was 6.25 Gy (Table 2).

The corresponding values, in the case of functional lung volume assessed excluding emphysematous lung areas according to a CT threshold, were 0.46% for FCL_V20, 56.7% for FCL_V5, and 6.35 Gy for FCL_MLD. Statistically significant differences were found when comparing conventional dose values with their functional counterparts, underlining the distinct impact of dose on functional lung volumes, particularly in V20, V5, and MLD values. During the post-treatment follow-up period, 42% of the patients developed acute radiation pneumonitis; of these, 28% exhibited symptomatology that was moderate-to-severe in nature (Grades 2–3), for which medical intervention was necessary. These usually presented with symptoms such as shortness of breath and dry cough, which were manageable with steroids and other symptomatic measures. Mild cases (Grade 1) were more common and generally resolved spontaneously. The association of a higher V20 in the contralateral lung was significantly related to the risk of developing pneumonia.

**Figure 1:**
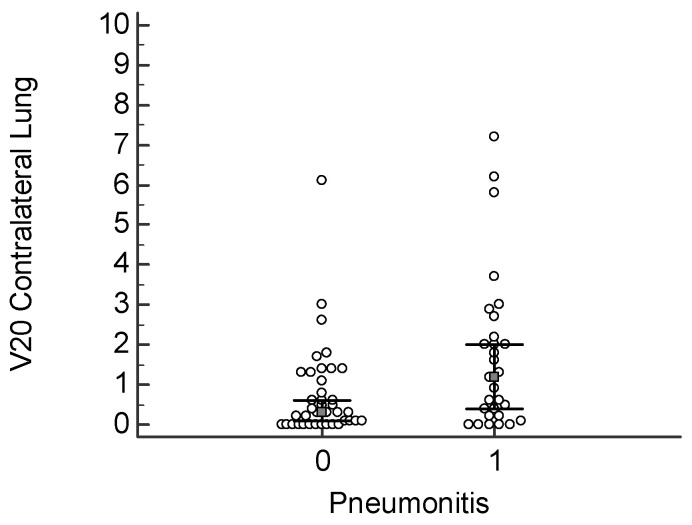
Distribution of V20 values in the contralateral lung in patients with and without radiation pneumonitis. Dot plot showing the V20 (%) for the contralateral lung among patients who developed (1) or did not develop (0) any grade of radiation pneumonitis. The group with pneumonitis exhibited a significantly higher median V20 (*p* = 0.017).

**Figure 2:**
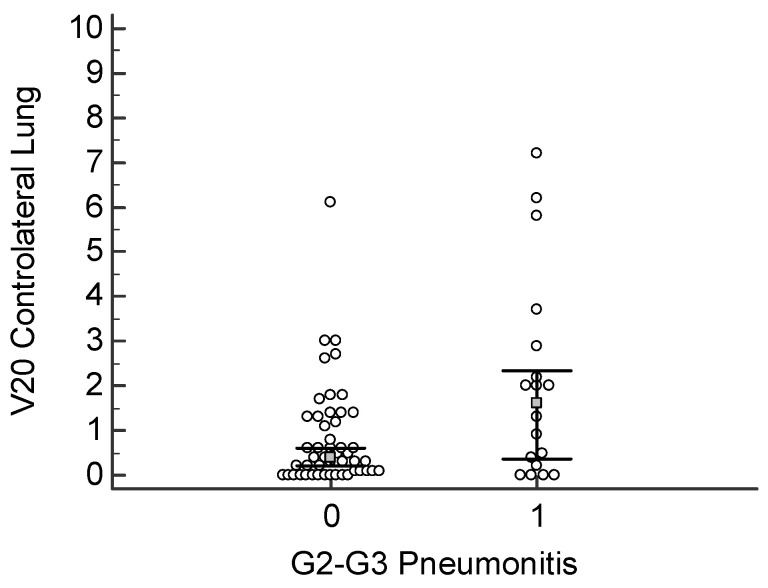
Distribution of V20 values in the contralateral lung according to pneumonitis severity. Dot plot comparing V20 (%) between patients with grade 0–1 versus grade 2–3 pneumonitis. A higher V20 was significantly associated with increased risk of moderate–to–severe pneumonitis (*p* = 0.028).

**Figure 3:**
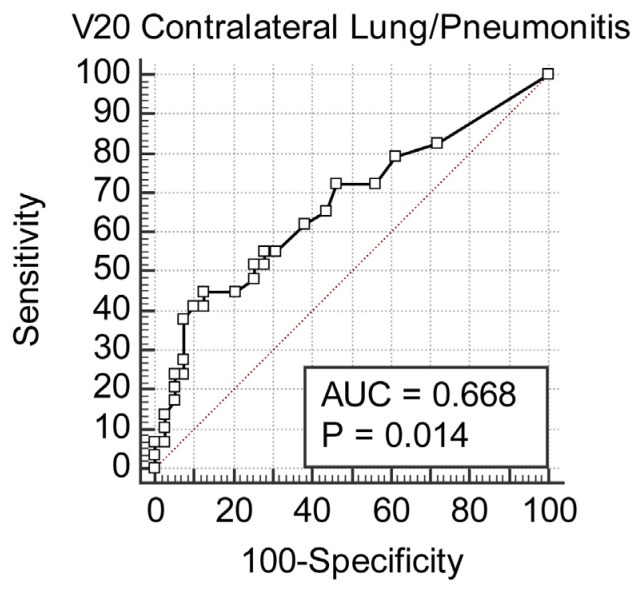
Receiver operating characteristic (ROC) curve for V20 predicting any grade of radiation pneumonitis. The ROC curve demonstrates the predictive ability of contralateral lung V20 for any-grade radiation pneumonitis, with an area under the curve (AUC) of 0.668 and *p* = 0.014.

**Figure 4:**
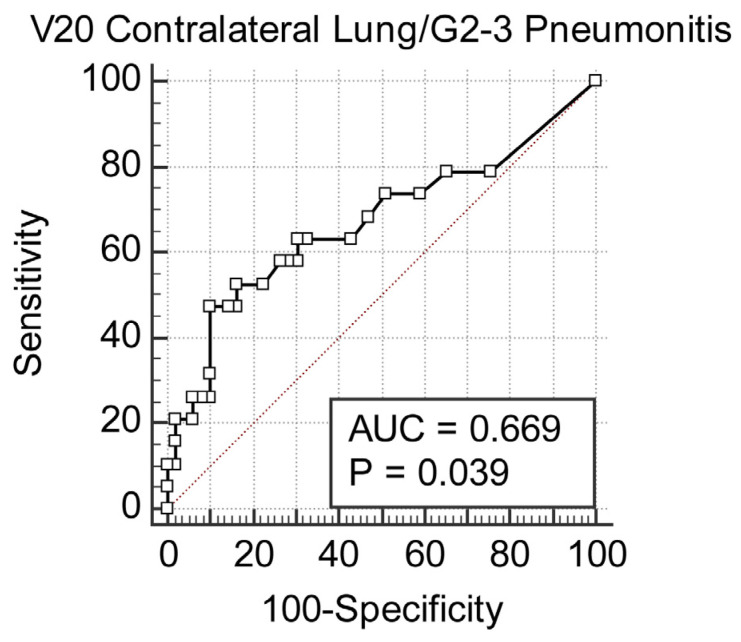
ROC curve for V20 predicting grade 2–3 radiation pneumonitis. The AUC for predicting moderate–to–severe pneumonitis (grade 2–3) using contralateral lung V20 was 0.669 (*p* = 0.039), indicating moderate discriminative performance.

A V20 value >1.2% significantly increases the risk for pneumonia; this correlation reached a statistical significance of *p* = 0.017 (Figure 1). In the same way, the FCL_V20 above 1% was significantly related to an increased risk of developing pneumonia, with *p* = 0.028 (Figure 2). The classic V20 metric, in its predictive performance, gave an AUC of 0.668, thus showing a moderate predictive power for pneumonia (Figure 3 and Figure 4). FCL_V20, although calculated with the intention to further refine the predictions by focusing on functional lung regions, has produced an AUC of 0.655. Statistical comparison showed no significant improvement over conventional V20, suggesting that though functional lung delineation indeed provides an alternative perspective, it does not necessarily increase predictive accuracy in this patient group. Among the parameters studied, only V20 showed a significant correlation for the prediction of more severe pneumonia (grade 2–3). The recommended threshold, according to the Youden index, was set at 1.8%. At this cutoff, a sensitivity of 47.3% and specificity of 89.8% were demonstrated, thus implying the marker’s potential for identifying patients at higher risk of developing severe pneumonia.

The total median contralateral lung volume was 1781 cc, whereas the median functional contralateral lung volume (FCL) was 1491 cc.

All patients reported their smoking status: 44 (56%) were non-smokers, 25 (32%) were ex-smokers, and 9 (12%) continued to smoke. Clinical TNM staging information was inconsistently available and therefore was not considered in the current study.

Pulmonary comorbidities such as COPD or emphysema were reported in 10 patients (13.2%) and were included in the descriptive analysis.

## 4. Discussion

Malignant pleural mesothelioma remains one of the major therapeutic challenges, owing to its aggressive nature and the anatomical intricacy of the thoracic region. The goal of the current study is adjuvant radiotherapy efficacy evaluation with a dose distribution analysis on the risk factor profile for lung toxicity, and RP in particular.

These results offer an optimistic view that RP risk may be mitigated by accurate dosimetric planning and the use of advanced radiotherapy techniques. Indeed, recent publications have pointed out positive data supporting such a view. Thompson et al. (2019) showed that V20 ≤ 25% and MLD below 14 Gy reduce the risk of RP while adequately covering the target volume [19]. Similarly, Zhang et al. (2012) emphasized that precise control of dosimetric parameters can limit the incidence of pulmonary toxicity, identifying MLD ≤ 14 Gy as a key value for prevention [20].

Compared to the work by Thompson et al. [19], which included heterogeneous thoracic oncology populations and a range of treatment techniques, our study specifically analyzes a homogeneous cohort of patients with malignant pleural mesothelioma (MPM) treated with IMRT after pleurectomy/decortication (P/D). Moreover, we directly compare anatomical and functional dosimetric parameters, introducing the FCL_V20 metric and evaluating its predictive role for radiation pneumonitis using ROC curve analysis. This refined approach, focused on functional lung volumes, aims to enhance risk stratification and treatment individualization in the MPM setting.

Uchida et al. presented a further development of predictive models by introducing the exclusion of emphysematous areas (LAV, Low Attenuation Volume), showing that the ratio between the non-emphysematous lung volume irradiated at ≥30 Gy and the total volume is a highly predictive indicator (AUC = 0.894) [21]. It constitutes a promising approach in decreasing toxicity even more.

The use of modern radiotherapy techniques such as IMRT and VMAT has demonstrated actual benefits. As Franceschini et al. (2020) noted, VMAT significantly reduces the risk of serious adverse effects but is still effective in treatment [22].

They also remarked on the fact that optimal management of heart dose indirectly contributes to reducing pulmonary toxicity. Kishan et al. (2015) compared IMRT with 3D-CRT and demonstrated that IMRT is associated with a more homogeneous dose distribution and better sparing of organs at risk [23].

Ozyurt et al. (2023) showed that the adoption of Helical TomoTherapy (HTT) is associated with excellent local disease control, with effective toxicity management, particularly in patients with reduced lung volumes [12]. Similarly, Parisi et al. (2023), in the MESO-RT trial, investigated hypofractionated schemes (30 Gy in 5 fractions with a selective boost up to 40 Gy), demonstrating significant logistical improvements with good patient tolerability [3].

It should be noted that although Trovò et al. (2019) note some cases of grave adverse events even in modern patients undergoing extrapleural pneumonectomy, these are now uncommon due to the advances in the planning and delivery of radiotherapy techniques [24]. Rosenzweig (2017) were able to confirm that continuing adherence to dosimetric constraints plays a further critical role in minimizing adverse events [25].

These observations were further solidified by Layer et al. (2024), who highlighted the predictive role of dosimetric parameters in toxicity outcomes [26]. Indeed, according to their analyses, strict adherence to dose constraints for critical organs significantly reduces pulmonary complications. Stahel et al. (2015) similarly reported that survival benefits from hemithoracic RT after extrapleural pneumonectomy are not significant, although careful patient selection and dosimetric management remain important [27].

These innovations only support the increasing use of precision medicine in MPM management. Better imaging, together with the latest advancements in delivery systems, has substantially enhanced our capacity to target tumor tissue while preserving healthy lung parenchyma. Such progress is all the more well-documented in trials like those by Uchida et al. (2017) and Franceschini et al. (2020), in which detailed dosimetric parameters and optimization of treatment planning were translated into clinical benefits [21,22].

The dose–volume parameter’s relationship with the risk of RP is currently well-established, and parameters beyond V20 to MLD were recognized as more modern metrics, with FLC_V20 and the exclusion of emphysematous lung areas for safer prediction and management of pulmonary toxicity. As determined by Zhang et al. in the year 2012, thresholds of greater than 14 Gy MLD and greater than 25% V20 are most strongly predictive for RP, delineating strict adherence to these threshold values [20].

### 4.1. Predictive Factors and Biomarkers

Biomarkers add further layers of risk stratification in clinical practice. Zhang et al. (2012) identified that increased post-RT plasma TGF-β1 levels are associated with a high risk for RP and thus may serve as an early biomarker for toxicity [20]. This is further emphasized by Kimura et al. (2012), who identified baseline pulmonary function, the presence of pre-existing emphysema, and comorbid conditions like diabetes and COPD as important factors in the identification of high-risk patients [17].

Biomarker integration holds particular promise in the setting of personalized medicine. The integration of dosimetric data with biomarkers such as TGF-β1 may permit clinicians to prospectively identify those patients at increased risk of RP and to modify treatment plans accordingly. This strategy not only minimizes toxicity but also maximizes the overall effectiveness of treatment.

### 4.2. Clinical Implications

These study results, taken in tandem with evidence from the literature, point toward a multidisciplinary approach in managing the risk for RP. Optimization of outcomes could be developed with dosimetric parameters and modern RT techniques combined with the integration of biomarkers. The techniques of IMRT, VMAT, and HTT revolutionized planning by offering excellent dose distribution, sparing healthy lung tissue from unwanted radiation burden [12,22,28,29]. These have been of great importance in bringing down the rates of RP and improving the outcome for patients. To this end, there is the need to retain dosimetric constraints: parameters should inform treatment planning, including V20 ≤ 25%, MLD ≤ 14 Gy, while accurate avoidance of emphysematous lung regions (LAVs) is ensured [19,20,30]. Also, predictive biomarkers introduced in clinical practice can improve risk stratification and, hence, enable timely intervention in high-risk patients [17,31].

Along these lines, our investigation corroborates the clinical importance of integrating functional lung dosimetry—particularly FCL_V20—within standard treatment planning. Improving precision in determining the risk of RP with this method can indeed help in safe radiation delivery in a particularly vulnerable group of patients, i.e., those with pre-existing pulmonary weakness. Such a functional-based strategy is a good addition to personalized approaches using dosimetry and biomarkers, thus advancing a more holistic precision medicine approach in MPM.

## 5. Conclusions

In the end, this study, among the referenced research works, evidenced the view that the optimization of dosimetric parameters such as V20, MLD, and FLC_V20, the incorporation of biomarkers like TGF-β1, and advanced techniques of radiotherapy such as IMRT, VMAT, and HT have become useful tools in reducing the chances of acute radiation pneumonitis. Predictive modeling, personalized medicine, and new technologies in patient treatment are most likely to yield the best results in making radiotherapy safer for patients with MPM. In fact, more improvement is needed, especially in future trials of these methods, to achieve the best treatment results.

## Figures and Tables

**Table 1:** Clinical characteristics of the patients included in the study, including age, gender, type of surgical resection, and chemotherapy regimen.

Characteristic	Details
Number of Patients	68
Median Age (years)	65
Age Range (years)	37–78
Gender Distribution	Majority Male
R0 Resection (%)	85% (58 patients)
R1 Resection (%)	15% (10 patients)
Chemotherapy Regimen	Platinum–pemetrexed
Number of Chemotherapy Cycles (median, range)	6 (range: 3–9)

**Table 2:** Dosimetric parameters related to radiotherapy treatment, with median values (range) and statistical significance (*p*-value), compared between patients with and without pulmonary toxicity.

Dosimetric Parameter	Median Value (Range)	*p*-Value
Total Dose (Gy)	44 Gy (50.6–59.4 Gy)	-
Boost Dose (Gy)	50.6 Gy (50.6–59.4 Gy)	-
V20 (Contralateral Lung)	0.5% (0.3–1.1%)	0.017
V5 (Contralateral Lung)	55% (48.7–57.7%)	0.97
Mean Lung Dose (MLD)	6.25 Gy (6.0–6.5 Gy)	0.98
FCL_V20 (Functional Lung)	0.46% (0.17–0.88%)	0.028
FCL_V5 (Functional Lung)	56.7% (49.9–59.5%)	0.55
FCL_MLD (Functional Lung)	6.35 Gy (6.03–6.75 Gy)	0.32

## Data Availability

The data presented in this study are available on request from the corresponding author. The data are not publicly available due to patient privacy restrictions.

## Abbreviations

The following abbreviations are used in this manuscript:

MPM	Malignant Pleural Mesothelioma
RP	Radiation Pneumonitis
P/D	Pleurectomy/Decortication
CT	Computed Tomography
FLV	Functional Lung Volume
V20	Volume of Lung Receiving ≥ 20 Gy
MLD	Mean Lung Dose
IMRT	Intensity-Modulated Radiotherapy
CTCAE	Common Terminology Criteria for Adverse Events
ROC	Receiver Operating Characteristic
AUC	Area Under Curve
FCL	Functional Contralateral Lung
